# The fungal composition of natural biofinishes on oil-treated wood

**DOI:** 10.1186/s40694-017-0030-5

**Published:** 2017-01-26

**Authors:** Elke J. van Nieuwenhuijzen, Jos A. M. P. Houbraken, Peter J. Punt, Guus Roeselers, Olaf C. G. Adan, Robert A. Samson

**Affiliations:** 1 Applied and Industrial Mycology, CBS-KNAW Fungal Biodiversity Centre, Utrecht, The Netherlands; 2 TNO, Microbiology and Systems Biology, Zeist, The Netherlands; 3 Dutch DNA Biotech BV, Zeist, The Netherlands; 4 Present Address: Danone Nutricia Research, Utrecht, The Netherlands; 5 Department of Applied Physics, Section Transport in Permeable Media, University of Technology Eindhoven, Eindhoven, The Netherlands

**Keywords:** Biofilm, Metagenomics, Mould, Wood protection, Wood staining

## Abstract

**Background:**

Biofinished wood is considered to be a decorative and protective material for outdoor constructions, showing advantages compared to traditional treated wood in terms of sustainability and self-repair. Natural dark wood staining fungi are essential to biofinish formation on wood. Although all sorts of outdoor situated timber are subjected to fungal staining, the homogenous dark staining called biofinish has only been detected on specific vegetable oil-treated substrates. Revealing the fungal composition of various natural biofinishes on wood is a first step to understand and control biofinish formation for industrial application.

**Results:**

A culture-based survey of fungi in natural biofinishes on oil-treated wood samples showed the common wood stain fungus *Aureobasidium* and the recently described genus *Superstratomyces* to be predominant constituents. A culture-independent approach, based on amplification of the internal transcribed spacer regions, cloning and Sanger sequencing, resulted in clone libraries of two types of biofinishes. *Aureobasidium* was present in both biofinish types, but was only predominant in biofinishes on pine sapwood treated with raw linseed oil. Most cloned sequences of the other biofinish type (pine sapwood treated with olive oil) could not be identified. In addition, a more in-depth overview of the fungal composition of biofinishes was obtained with Illumina amplicon sequencing that targeted the internal transcribed spacer region 1. All investigated samples, that varied in wood species, (oil) treatments and exposure times, contained *Aureobasidium* and this genus was predominant in the biofinishes on pine sapwood treated with raw linseed oil. *Lapidomyces* was the predominant genus in most of the other biofinishes and present in all other samples. Surprisingly, *Superstratomyces*, which was predominantly detected by the cultivation-based approach, could not be found with the Illumina sequencing approach, while *Lapidomyces* was not detected in the culture-based approach.

**Conclusions:**

Overall, the culture-based approach and two culture-independent methods that were used in this study revealed that natural biofinishes were composed of multiple fungal genera always containing the common wood staining mould *Aureobasidium*. Besides *Aureobasidium*, the use of other fungal genera for the production of biofinished wood has to be considered.

**Electronic supplementary material:**

The online version of this article (doi:10.1186/s40694-017-0030-5) contains supplementary material, which is available to authorized users.

## Background

**Fig. 1:**
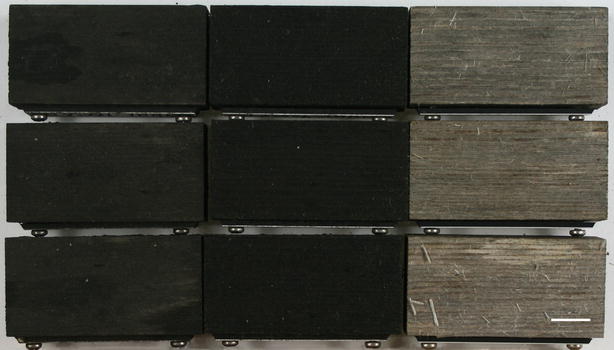
Oil-treated pine sapwood samples with natural formed biofinish and weathered samples without biofinish. Samples in the *left row*: treated with raw linseed oil. Samples in the *middle row*: treated with olive oil. Samples in the *right row*: untreated. *Scale bar* 10 mm

Microbial growth causing discolouration on surfaces of outdoor situated materials is a common phenomenon [1–3]. Frequently these microbial stains are referred to as biofilm, although not all commonly accepted biofilm criteria might have been investigated [4]. Dark staining of painted and unpainted wood is mostly attributed to fungi and generally considered as unwanted discolouration [5, 6]. In contrast, the specific dark stain formation on wood called biofinish is considered to be a functional colouration [4] (Fig. 1). The colouration of a biofinish is, together with its presumed protection and self-healing properties, an important ingredient of a sustainable solution for a biocide free wood finish system [4, 7]. A biofinish refers to a dark pigmented layer, that covers a wood surface almost entirely without exposing underlying wood structures, contains abundant microbial mass and that is irreversibly attached to the surface [4]. Biofinishes have been detected on wood impregnated with olive oil or raw linseed oil [4, 8]. The study described in this paper is focused on the characterization of the fungal composition of these biofinishes.

Although several fungal species are associated with outdoor wood staining [6, 9], little is known to which extent each taxon contributes to this staining. In some studies the fungal populations on timber surfaces were quantified [10, 11]. The study by Sailer et al. [7] provides data, particularly of interest for the biofinishes on wood. One specific sample, made of pine sapwood and impregnated with refined linseed oil dissolved in acetone, was used to study the fungal composition of a homogenous dark stained wood surface. Later, biofinishes were detected on other outdoor exposed oil-treated wood samples, including samples made of different wood species treated with olive oil. Analysis of these samples revealed the abundancy of the wood staining fungus *Aureobasidium*, but the results also indicated that this genus might not always dictate the fungal population of dense dark stained wood samples [4, 12]. The wood species, oil type and the geographical location could influence the fungal community composition of a biofinish. In order to manufacture a stable biofinish and eventually apply biofinished wood in practice, a detailed composition of fungi present at various stained wood surfaces is elucidated in this paper.

Different techniques are available to study a fungal community on the surface of an environmental sample such outdoor exposed wood. Each technique has its advantages and disadvantages. Microscopic examination of the surface can be an easy method to study the surface of a material [13]. The main disadvantage is that identification and enumeration is difficult in case of moulds on oil-treated wood surfaces [4]. A common way to identify and enumerate fungi is by culturing. A swab-based method can be used to analyse the culturable fungi that are present on a sample surface [4, 14]. This includes the determination of the number of colony forming units after incubation and identification based on macroscopic, microscopic and/or molecular analysis. Culture-based analysis only allows the detection of readily culturable species, which is effected by the media selection and overestimates the presence of abundantly sporulating species [13]. Culture-independent methods based on DNA analysis are frequently used and show complementary results [15–17]. Culture-independent approaches that rely on Next Generation Sequencing (NGS) methods have become state of the art to study microbial communities [16, 18, 19]. Albeit that these NGS methods provide advantages compared to earlier developed techniques, research to control and understand the biases occurring in all steps of a NGS method is still ongoing [18–20].

The objective of the present study was to analyse the fungal composition of various biofinishes on oil-treated wood surfaces. A culture-based swab method and two non-culturing methods based on either amplicon-cloning followed by Sanger sequencing or Illumina amplicon sequencing were selected for the analysis. Wood samples without oil treatment and/or biofinish were used to compare the diversity and predominance of fungal genera.

## Methods

Several natural biofinishes were studied with a culture-based and two DNA sequencing-based methods (Table 1). The biofinish containing samples varied in wood species, type of oil and origin. Wood samples made of the same wood species without a biofinish, were studied as well. The viable fungal composition was studied of all samples. Specific samples, which were exposed in the Netherlands, were selected for the culture-independent fungal profiling methods.

**Table 1:** Overview of the amount and type of wood samples used per type of fungal profiling method

Sample set	Wood species	Treatment	Presence biofinish	Location (exposure time)	Number of samples		
					Culture method	Cloning method	Illumina method
1	Spruce	Raw linseed oil	No	Utrecht, The Netherlands (1.5 year)	3		1
		Stand linseed oil	No		3		1
		Olive oil	Yes^a^		3		1
		No oil	No		3		1
	Pine sw	Raw linseed oil	Yes^a^		3	3	2
		Stand linseed oil	No		3		1
		Olive oil	Yes^a^		3	3	2
		No oil	No		3		1
	Ilomba	Raw linseed oil	No		3		1
		Stand linseed oil	No		3		1
		Olive oil	Yes^a^		3		1
		No oil	No		3		1
2	Pine sw	Raw linseed oil	Yes^a^	Utrecht, The Netherlands (1.8 year)	10		2
3	Spruce	Raw linseed oil	No	Utrecht, the Netherlands (1. 5 year)	1		
	Ilomba	Raw linseed oil	No		1		
	Pine sw	Raw linseed oil	Yes^b^		1		
	Pine sw	Olive oil	Yes^b^		1		
	Pine sw	No oil	No		1		
	Pine hw	Raw linseed oil	Yes^b^		1		
4	Same materials as set 3		Biofinish only on olive oil	Johannesburg, South Africa (1.7 year)	1		
5	Same materials as set 3		No	Dover Gardens, Australia (1,5 year)	1		
6	Same materials as set 3		Biofinish on pine and r. lins.	Ås, Norway (2 year)	1		

^a^Biofinish assessment by Nieuwenhuijzen, van et al. [4]

^b^Determination based on stain coverage

### Wood samples

The different wood species tested were pine (*Pinus sylvestris*), spruce (*Picea abies*) and ilomba (*Pycnanthus angolensis)*. Pine samples were made totally of sapwood (sw) or a mixture of sapwood and heartwood (hw). No specific sapwood or heartwood selection was made for spruce and ilomba. Wood blocks were impregnated with raw linseed, stand linseed or olive oil. Sets of impregnated and untreated wood samples were exposed outdoors at different locations (Table 1). The sample dimensions, oil treatments, outdoor exposure and handling procedures were described in van Nieuwenhuijzen et al. [4]. All samples, except for the samples of set 3, have been analysed with the biofinish assessment method. This method consists of observations of the dark stained surface coverage at macroscopic and microscopic scale, and spectrophotometer measurements of the pigmentation [4]. In summary, a biofinish is assigned when more than 90% of the surface is stained and does not expose structures of the wood such as annual rings or wood fibres, and the pigmentation measurements, expressed by sRGB colour space triplets, meet specific criteria (R, G and B values are below 82 and the value difference within a single RGB triplet is below 20). The presence of biofinishes on the wood samples of set 3 was only estimated with visual observations of the stain coverage as described in the biofinish assessment method, which can overestimate biofinish identification.

### Culturing colonies

The concentration of colony forming units (CFU) per cm^2^ wood was determined for each specimen (Table 1). For this, biomass collected with a cotton swab was analysed as described by van Nieuwenhuijzen et al. [4]. Serial dilutions were plated in duplicate (set 1 and 3–5) or triplicate (sample set 2) on dichloran 18% glycerol agar (DG18) and malt extract agar (MEA) supplemented with penicillin and streptomycin (P/S). The agar media was prepared as described by Samson et al. [13]. The total number of colonies and those phenotypically resembling *Aureobasidium* was determined after seven and fourteen days of incubation at 25 °C [12]. Besides the *Aureobasidium* colonies, also the predominant colonies were counted. Two or more colonies of each predominantly present colony type were transferred to new MEA plates. The isolates were deposited in the working collection of the Applied and Industrial Mycology department (DTO) housed at the CBS-KNAW Fungal Biodiversity Centre, The Netherlands and subjected to molecular identification.

### DNA extraction

DNA was extracted from cultures grown on MEA plates according to van Nieuwenhuijzen et al. [12]. With respect to the culture-independent methods, biomass was removed with a sterile scalpel from the upper surface of a mould stained wood sample, collected on sterile paper and subsequently used to extract DNA [4]. In both cases the Ultraclean Microbial DNA isolation kit (MoBio Laboratories, USA) was used according to manufacturer’s instructions.

### PCR and Sanger sequencing of isolates

The nuclear internal transcribed spacers including the 5.8S rRNA gene (ITS) of fungal isolates were amplified with the primer pair V9G [21] and LS266 [22]. In case additional sequence information was needed for a proper identification of a strain, the *LSU* gene was partially amplified using the forward primer LROR (Reh) GTACCCGCTTGAACTTAAGC [23] or LROR (VilU) ACCCGCTGAACTTAAGC [Vilgalys, unpublished] and the reverse primer LR5 or LR7 [24]. The polymerase chain reaction (PCR) mixtures had final concentrations of: 4% DNA extract, 10% PCR buffer, 3% MgCl_2_ (25 mM), 65.8% demineralised sterile water, 7.8% dNTP (1 mM), 5% DMSO, 2% forward primer (10 μM), 2% reverse primer and 0.4% Taq polymerase (5 U/mL, BioTaq, Bioline). The PCR program typically consisted of 1 cycle of 5 min denaturation at 95 °C; 35 cycles of 35 s denaturation at 95 °C, followed by ITS-primer annealing for 30 s at 55 °C or *LSU*-primer annealing at 54 °C for 50 s, and an extension for 1.5 min at 72 °C. The PCR-products were sequenced with the same primers as used for PCR amplification using the BigDye Terminator v. 3.1 Cycle Sequencing Kit (Applied Biosystems, USA). Sequence products were analysed on an ABI PRISM 3730XL genetic analyser (Applied Biosystems, USA) and traces were assembled using Seqman Pro v. 9.0.4 (DNAstar Inc.). The sequences were deposited in GenBank [25].

### PCR, cloning and Sanger sequencing

ITS-specific clone libraries were made as described in van Nieuwenhuijzen et al. [12] of two types of biofinishes, formed on different substrates exposed in the Netherlands (Table 1, set 1): pine sapwood treated with raw linseed oil (libraries PRL.1, PRL.2 and PRL.3) and pine sapwood treated with olive oil (PO.1, PO.2 and PO.3). The ITS region was amplified with the primers V9G and LS266 and the GoTaq Long PCR Master Mix (Progema), while using the PCR-program as described above. Purified PCR products (QIAquick PCR purification kit) were ligated and cloned (pGEM^®^-T Easy Vector Systems) into an *Escherichia coli* plasmid library. Amplification and Sanger sequencing of DNA from ITS containing competent cells was performed as described in van Nieuwenhuijzen et al. [12]. The sequences were deposited in GenBank [25].

**Table 2:** The predominantly cultured colony types of each wood sample set

Sample set	Wood species	Treatment	No. of sam-ples	Biofinish	Number of wood samples with predominant colony types											
					*Aureobasidium*	Black yeasts	*Cladosporium*	*Cryptococcus*	*Didymellaceae*	*Phacidiella*	*Pleurophoma*	*Pyrenochaeta*	*Cyanodermella*	*Superstratomyces*	*Sydowia*	*Taphrina*
1	Spruce	R. lins. oil	3	No	3	1										
		St. lins. oil	3	No	3	3										
		Olive oil	3	Yes^a^	2									3		
		No oil	3	No	1									3		
	Pine sw	R. lins. oil	3	Yes^a^	1	1								2		1
		St. lins. oil	3	No		3								3		
		Olive oil	3	Yes^a^										3		
		No oil	3	No		2								3		
	Ilomba	R. lins. oil	3	No					1					2		
		St. lins. oil	3	No		2	1							2		
		Olive oil	3	Yes^a^										3		
		No oil	3	No			3				1			1		
2	Pine sw	R. lins. oil	10	Yes^a^	3	1				1		1		10		2
3	Spruce	R. lins. oil	1	No										1		
	Ilomba	R. lins. oil	1	No										1		
	Pine sw	R. lins. oil	1	Yes^b^	1									1		
	Pine sw	Olive oil	1	Yes^b^										1		
	Pine sw	No oil	1	No										1		
	Pine hw	R. lins. oil	1	Yes^b^	1											
4	Spruce	R. lins. oil	1	No	1											
	Ilomba	R. lins. oil	1	No	1											
	Pine sw	R. lins. oil	1	No	1											
	Pine hw	R. lins. oil	1	No	1	1										
5	Spruce	R. lins. oil	1	No	1											
	Ilomba	R. lins. oil	1	No	1	1										
	Pine sw	R. lins. oil	1	No	1											
	Pine sw	Olive oil	1	No		1		1								
	Pine sw	No oil	1	No	1											
	Pine hw	R. lins. oil	1	No	1								1			
6	Spruce	R. lins. oil	1	No											1	
	Ilomba	R. lins. oil	1	No		1									1	
	Pine sw	R. lins. oil	1	Yes^a^											1	
	Pine sw	Olive oil	1	No											1	
	Pine sw	No oil	1	No											1	
	Pine hw	R. lins. oil	1	Yes^a^		1									1	

^a^Biofinish detection by van Nieuwenhuijzen et al. [4]

^b^Determination based on stain coverage

### Illumina ITS1 amplicon sequencing

Internal transcribed spacer 1 region (ITS1) amplicon libraries were made of 16 wood samples that were all exposed at one test site (The Netherlands), but variated in wood species, treatment and the presence or absence of a biofinish (Table 1, set 1–2). In a preliminary study several ITS primer combinations and amplification approaches were tested for their suitability of generic detection of fungal genera (unpublished results). Based on these results barcoded ITS1 amplicons were generated using a two-step PCR approach. ITS1 regions were first amplified with the following primers: nex-ITS-BITS-F: TCGTCGGCAGCGTCACCTGCGGARGGATCA and nex-ITS-B58S3-R: GTCTCGTGGGCTCGGGAGATCCRTTGYTRAAAGTT (adapted from Bokulich and Mills [26]). Each reaction contained 300× purified DNA, 1X hot start PCR master mix (Thermo Scientific) and nuclease free PCR grade water to a 50 µl final reaction volume. PCR reactions consisted of an initial denaturation step of 95 °C for 5 min and 30 amplification cycles (95 °C for 30 s, annealing 52 °C for 45 s and elongation 72 °C for 1 min) and a final extension step (72 °C for 10 min) followed by cool down (10 min at 4 °C). A negative control (blank) was included for each 24 PCR reactions. Reactions were cleaned by solid-phase reversible immobilization (SPRI) using AMPure XP SPRI beads (Beckman Coulter, Inc.). Dual barcodes (8 bp) and Illumina Sequencing adapters were attached using the Nextera XT Index Kit (Illumina, San Diego, CA) according to manufacturer’s protocols. Barcoded amplicons were quantified using the Caliper LabChip GX II system (Perkin Elmer, Hopkinton, USA), normalised to the same concentrations, pooled, and gel purified using the Qiaquick spin kit (Qiagen) and AMPure XP SPRI beads. Pooled amplicons were 250-bp paired-end sequenced using the MiSeq system (Illumina). Raw Illumina fastq files were deposited in the European Nucleotide Archive (accession PRJEB13755). The raw data was demultiplexed, quality filtered, and analysed using modules implemented in the Mothur software platform [27]. Quality filtering involved a minimal quality score of 25 in a window of 5 and removal of reads with a length below 100 and above 500 or with ambiguous bases. In addition, chimeras detected using UCHIME and the UNITE database (v6) [28] were removed. Filtered raw reads were merged into paired reads. Subsequently, the relative abundance of unique sequences were calculated for each sample by dividing the number of reads of a single unique sequence by the total number of reads of the sample. Unique sequences with a total sum of relative read abundancies above 0.1% were used for further analysis.

### DNA data analysis

ITS and ITS1 sequences were subjected to nucleotide BLAST searches [29] using the non-redundant database of GenBank [25], the Q-bank Fungi database [30] and an internal database of the CBS-KNAW Fungal Biodiversity Centre (Fungal Barcoding data). Identification was performed on genus level. ITS sequences obtained from non-cultured material, which resulted in hits in GenBank with an identity below 97% were marked as ‘unidentified’. Also the ITS1 sequences with query coverages below 90% were marked as ‘unidentified’. For the identification of culturable isolates with an unclear identity based on only ITS sequences, *LSU* sequences were also compared with the non-redundant nucleotide database of GenBank. ITS sequences of isolates that could not be identified on genus level were aligned with the sequences of the ITS cloning and ITS1 amplicon libraries using Nucleotide BLAST of GenBank(NCBI, Rockville Pike, USA).

### Mathematical analysis

A Shannon’s diversity test was used to index the genus diversity in the Illumina amplicon data.

## Results

### Culturable fungal composition

After culturing biomass of 70 wood samples, the predominantly colony types of 68 samples could be identified (Table 2). The total number CFU’s per square centimetre biofinish varied between 6 × 10^2^ and 4 × 10^5^ CFU/cm^2^ and the samples without biofinish, which all contained dark mould stains but less than the biofinish criteria prescribed, showed similar results (2 × 10^1^ to 4 × 10^5^ CFU/cm^2^; Additional file 1: Table S1). Frequently, more than one predominant colony type could be determined in the plated biomass from a single wood sample (Table 2). The isolates and sequence data obtained to identify the predominant type of CFU are listed in Additional file 2: Table S2.

**Fig. 2:**
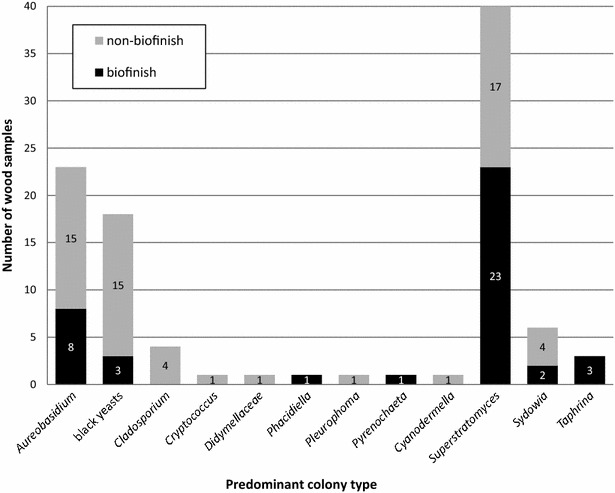
Identified colony types predominantly isolated from 28 outdoor exposed wood samples with a biofinish

A recently described dark pycnidia producing coelomycete named *Superstratomyces* was detected as the most commonly occurring predominant colony type in the biofinishes (Table 2; Fig. 2) [31]. This genus was also predominantly present on outdoor exposed samples without a biofinish, including samples without oil (Table 2). Interestingly, the detection of *Superstratomyces* on wood was restricted to samples exposed in the Netherlands (sample set 1–3).

*Aureobasidium* was predominantly present on 8 of the 28 samples that showed a biofinish (Table 2; Fig. 2). Also samples without a biofinish frequently showed this genus to be one of the predominant colony types (Table 2; Fig. 2). *Aureobasidium* was isolated from samples existing of all combinations of wood species and (oil) treatments originating from all selected outdoor locations (Additional file 1: Table S1, Additional file 2: Table S2). The *Aureobasidium* contribution to the total cultured CFU varied largely for biofinish samples (0–97%), but the contribution per sample surface area only rarely (2 out of 28 samples) exceeded the 50% (Additional file 1: Table S1). The stained samples without a biofinish showed a similar range of percentages (0–97%), and the *Aureobasidium* contribution exceeded the 50% regurlary (13 out of the 42 samples; Additional file 1: Table S1).

Other colonies types which were predominantly isolated from samples with a biofinish were black yeasts (identfified as *Exophiala*, *Phaeococcomyces* or *Knufia*), *Taphrina*, *Sydowia*, *Phacidiella* and *Pyrenochaeta*. Black yeasts and *Sydowia* were also isolated as predominant colony types from the stained samples without a biofinish, expended by the genera *Cladosporium*, *Cryptococcus, Pleurophoma* and *Cyanodermella,* and a genus in the family *Didymellaceae* (Table 2).

### Fungal composition of ITS clone libraries

**Fig. 3:**
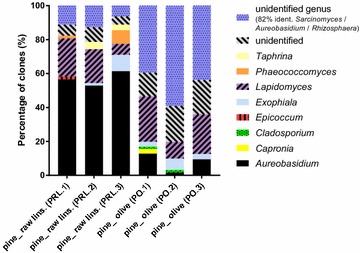
Genus composition in ITS clone libraries constructed from biofinish DNA. ‘Unidentified’: sequences resulting in best hits with an identity below 97%

Six ITS clone libraries were constructed from biofinish DNA obtained from pine sapwood samples treated with either raw linseed or olive oil. Each library contained 61–71 clones (Additional file 3: Table S3). In all libraries several genera were identified always including *Aureobasidium* (Fig. 3).

*Aureobasidium* was predominantly present in the cloned DNA of biofinishes on pine sapwood treated with raw linseed oil. In each of the three clone libraries more than 50% of the clones were identified as *Aureobasidium*. The investigated biofinishes on pine sapwood treated with olive oil did not show this predominance. Their clone libraries had a much lower *Aureobasidium* percentage, varying from 2 to 13%. Interestingly, the number of sequences which could not be assigned to the genus level was high in these samples. These divergent sequences all differed from the molecular identified strains obtained with the culturing method. Many of them had more than one best hit with sequences in the database while showing identity scores of 82% compared to sequences in GenBank that were named *Aureobasidium*, *Sarcinomyces* and *Rhizosphaera*. The other divergent sequences showed identity coverages varying from 80 to 96% compared to the best hits, which represented up to five genus names for each library (Additional file 3: Table S3). Furthermore, analysis of sequences obtained from all biofinish samples revealed the presence of other dark pigmented fungi, with *Lapidomyces* as a major contributor (6–27% per library). The in general more sparsely occurring genera were *Capronia, Cladosporium*, *Exophiala*, *Phaeococcomyces* and *Epicoccum*.

### Fungal composition of Illumina ITS1 libraries

**Fig. 4:**
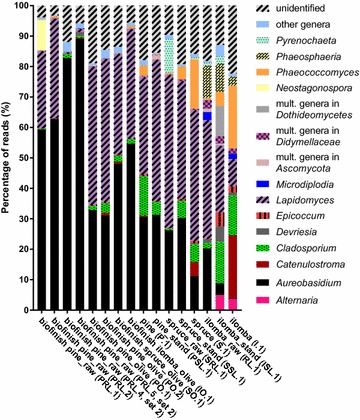
Genus composition in Illumina ITS1 amplicon libraries constructed from DNA of fungal stained wood surfaces. ‘Unidentified’: sequences resulting in best hits with an identity below 97% or a query coverage below 90%

**Fig. 5:**
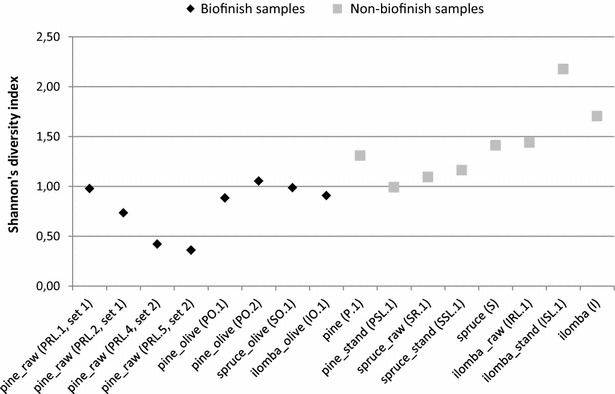
Shannon diversity index of the genus diversity in the Illumina data of wood samples with and without a biofinish

Application of the Illumina amplicon method to analyse the fungal biofinish community of the 16 selected wood samples resulted in a total of 2.17 million filtered ITS1 reads. The amount of reads passing the occurrence threshold was 2.02 million. Each wood sample had 6.5 × 10^4^ up to 4.6 × 10^5^ reads with a mean length of 171 nucleotides. In total 400 unique sequences were detected with no nucleotide variation among the reads of a single unique sequence (Additional file 4: Table S4). Most of these unique sequences could be identified to genus-level, but some sequences represented multiple genera or represented an unidentified genus (Fig. 4; Additional file 4: Table S4). The read percentage of this latter category varied for the biofinish samples between 3% till 22% of the total reads. Some of these sequences showed 100% similarity with the unidentified sequences obtained from the clone libraries, while none of these sequences were highly similar to the ITS sequences derived from any of the fungal isolates cultured from biofinishes. The number of different identified genera for the biofinish samples ranged from 26 to 34 and for the non-biofinish wood samples (that all contained dark mould stains but less than the biofinish criteria prescribed) from 27 to 35. The calculated Shannon’s diversity indices were generally lower for the biofinish samples (average 0.8) compared to the samples without a biofinish (average 1.4; Fig. 5).

The Illumina amplicon method revealed the presence of two predominant genera in the amplicon sequencing libraries of the eight samples that contained a biofinish: *Aureobasidium* and *Lapidomyces* (Fig. 4). Both genera were determined in the DNA extractions of all samples. In the amplicon sequence libraries of six biofinish containing samples the predominance of *Aureobasidium* was determined, including all four pine sapwood samples treated with raw linseed oil. These four samples had the highest contribution of *Aureobasidium* reads per sample (more than 56% for each sample). However, the predominance of this genus was only determined for half of the olive oil-treated samples that contained a biofinish. The other half of the samples contained *Lapidomyces* as the predominant genus. With respect to the analysed wood samples without biofinish, not one sample showed the predominance of *Aureobasidium* in the amplicon library, while most of them had *Lapidomyces* as predominantly present genus. In line with these results the percentage of *Aureobasidium* reads was higher for the biofinish samples [average 58%, standard deviation (SD) 20%] than for a non-biofinish sample (average 19%, SD 12%), despite the variation in substrates (Fig. 4). The two types of oil that were used for biofinish substrates differed in the percentages of *Aureobasidium* sequences. The biofinishes on wood treated with olive oil showed a lower percentage for *Aureobasidium* (31–55%) than the biofinishes on raw linseed oil (59–89%).

Although *Cladosporium* was detected in all amplicon sequencing libraries of the biofinish samples, the results showed a relative low contribution (average 1%, SD 1%) of this genus to the total reads of a library. The contribution of *Cladosporium* to the amplicon sequence libraries of samples without a biofinish was higher (average 7%, SD 5%). Similar results were found for the black yeast *Phaococcomyces* (biofinishes: average 0.2%, SD 0.2%; non-biofinishes: average 6%, SD 7%).

## Discussion

### The fungal composition of biofinishes

The fungal compositions generated with the culture-based, cloning and Illumina sequencing approach showed overlapping and partly complementary results. For example *Aureobasidium* was detected in biofinishes with all three techniques. In contrast, the genus *Superstratomyces*, detected by culturing as a predominant colony type, was absent in the data generated with the culturing-independent approaches. The monotypic genus *Lapidomyces*, represented by the rock-inhabiting species *L. hispanicus* [32], was detected in high numbers in the cloning and Illumina libraries, while it was absent in the predominantly cultured isolates. The culturing-independent approaches generated overlapping results, since *Aureobasidium* was detected with both techniques as predominant genus in the biofinishes on pine treated with raw linseed oil from set 1, with *Lapidomyces* as the secondly abundant genus. However, the results generated by these two methods of the biofinishes on pine sapwood treated with olive oil from set 1 were more complementary. Although both methods determined the presence of *Lapidomyces*, it was only predominant in the Illumina amplicon libraries, while the clone libraries had a remarkable high number of unidentified genera. In general the Illumina approach showed a larger diversity of genera (up to 30) compared to the clone approach (up to 7). Despite the complementary results of the three used techniques, each technique showed that biofinishes contain several genera including *Aureobasidium*.

The variation in the composition of the fungal community on a specific habitat with culture-dependent and culture-independent methods has been frequently reported [17, 33, 34]. Because each method is selective, variation in results seems inevitable. Firstly, only viable fungal propagules that are able to grow in specific lab conditions are identified in the culturing method, while in the case of the methods based on direct DNA extractions also non-culturable fungi can be detected. This explains why *Lapidomyces*, detected as one of the predominant genera in biofinishes, could only be found with the culture-independent techniques. One of the characteristics of this genus is the slow growth at low temperatures, such as 6 or 15 °C, and its inability to grow at 24 °C [32], while the incubation temperature used in this study was above 24 °C. Secondly, although a DNA-based method seems more complete than a culture-based, selection may happen already during the DNA extraction, since there is no equal efficiency of DNA extraction between all fungal species and/or cell structures [35, 36]. Also primers and PCR programs are known to selectively influence the profile of the microbial community [18, 19, 26]. Besides selection during DNA extraction and amplification, the possibility of variation in community composition due to low sampling numbers should be recognised in the case of the labour intensive cloning approach. Namely, the highest number of identified ITS-fragments represented not more than (71 clones/6,25 cm^2^ sampling area =) 11 fungal units per cm^2^ biofinish, while based on the CFU count the numbers in this study, only the culturable fungi can already be up to 4 × 10^5^ fungal units per cm^2^.

Fungal identification in this study was primarily based on ITS sequences and therefore restricted to genus level. The ITS locus, the formal fungal barcode [28, 37], is not necessarily unique for each species [38, 39] and particularly when only the ITS1 region is analysed. In case of *Aureobasidium*, the ITS sequences obtained from type strains [12] do show differences between species, but not when the ITS1 sequences are compared. Besides limited species discrimination, the taxonomic reliability of ITS sequences in a database can also be questioned [40], especially when updates of the data based on modern taxonomic revisions within, such as proposed for *Aureobasidium* [41], are lacking. In order to obtain more accurate species identifications multi-locus sequencing should be applied [12].

### The wood staining fungi *Aureobasidium*, *Lapidomyces* and *Superstratomyces* mainly contributed to the fungal biofinish composition

*Aureobasidium* was frequently detected with all tree techniques as predominant genus in natural biofinishes on oil-treated wood, which indicates the importance of this genus. Although the culturing method also revealed *Aureobasidium* among the predominant isolates of the stained wood samples that did not meet the specific biofinish criteria, the results of the Illumina approach used in this study showed that this predominance is not as easily detected as it may seem. All eight selected samples without a visible biofinish contained *Aureobasidium* in their amplicon library, but did not reveal its predominance , whereas six of the eight biofinishes did contain *Aureobasidium* as predominant detected genus. A few other fungal quantification studies of outdoor substrates, concerning the surface population of grapes, leaves of grapevines, apples and plasticised polyvinyl chloride, also indicate *Aureobasidium* to be predominantly present [42–45]. Other studied substrates, such as decomposing spruce logs [46], Scots pine needles [47], leaves/leaf litter [48], residential surfaces [49], public restroom floors [50] and the oral microbiome [51] contain *Aureobasidium,* but not as predominant fungus. In various substrates, even in fungal populations present in wood [52–55], *Aureobasidium* was not detected at all [33, 34, 56–59]. Apparently substrates and exposure conditions are selective and the surface of outdoor situated oil-treated wood samples that enables biofinish formation has favourable conditions for *Aureobasidium*.

The importance of genera other than *Aureobasidium* to outdoor biofinish formation has to be considered, especially of *Superstratomyces* and *Lapidomyces*. As expected from the previous results in the study by van Nieuwenhuijzen et al. [4], *Aureobasidium* was not always detected as the predominant genus of a biofinish. The culture-based method of the current study enabled the identification of at least five other predominant genera (Fig. 2) with *Superstratomyces* as the most commonly occurring predominant colony type isolated from biofinishes. The detection of *Superstratomyces* at outdoor exposed untreated wood samples showes that the presence of this genus is not limited to biofinishes or oil-treated substrates. In the clone libraries as well as the amplicon sequence libraries of biofinishes on pine sapwood treated with raw linseed oil, the predominance of *Aureobasidium* was determined. This is in line with the earlier finding of *Aureobasidium* as the only predominant genus in the fungal DNA extracted from a single specific sample with homogeneous dark staining published by Sailer et al. [7]. However, biofinishes on pine treated with olive oil contained more *Lapidomyces* than *Aureobasidium* sequences in their clone and Illumina amplicon libraries. The Illumina amplicon approach resulted in *Lapidomyces* as predominant genus, while sequences of an unidentified genus (top hits: *Sarcinomyces*/*Aureobasidium*/*Rhizosphaera*, maximum identity scores: 82%) were predominant in the clone libraries of these biofinishes.

In contrast to the potential importance of abundantly present genera in biofinishes, the importance of some of the detected genera can be questioned. For example, the results of the Illumina amplicon approach indicated a lower contribution of *Cladosporium* reads to the libraries of biofinish samples compared to the libraries of stained samples that did not meet the biofinish criteria(7%), but also a relative low abundance in total (1%). Also the black yeast *Phaeococcomyces* was far less represented in the libraries of samples with a biofinish than in the libraries of wood samples without a biofinish. These results are in line with the calculated Shannon’s diversity indices that indicated a lower diversity in the fungal population of biofinish samples than of wood samples without a biofinish.

### Competitive advantage of *Aureobasidium* in natural biofinishes

The detection of the structural presence and frequent predominance of *Aureobasidium* in biofinishes analysed in this study, confirms the important role of *Aureobasidiu*m in biofinish formation. As concluded from van Nieuwenhuijzen et al. [12] the development of *Aureobasidium* on oil-treated wood starts in the first few days of outdoor exposure. Since many parameters define the conditions on the surface of a material outdoors, it is likely that multiple factors are further responsible for the establishment of *Aureobasidium* in biofinishes on oil-treated wood.

In particular the production of melanin seems to be involved in the survival of *Aureobasidum* in biofinishes during outdoor exposure. Melanin plays a role in the protection against UV-light and other environmental stresses [60, 61]. Melanin production is observed in cultures of *Aureobasidium* [62, 63]. However, other genera are also known to produce melanins [60, 61]. Also the results in this study showed the presence of several other melanin producing fungi on weathered wood surfaces such as *Cladosporium* and *Exophiala*. Correlations between the pigmentation of fungal genera, the type and amount of melanins and their specific protective functions need to be investigated to understand the competitive advantage of *Aureobasidium*.

Also the presence of oil in wood, an essential ingredient for biofinish formation, is thought to play an important role at least due to its provision of carbon sources for *Aureobasidium* growth ([12], unpublished data). The results of the Illumina approach indicated that the addition of oil to a specific wood species is related to an increase in the amount of *Aureobasidium* biomass on the wood surface. However, the use of oil or it derivatives as nutrient for growth is also known for species of other genera such as *Exophiala* (unpublished data) and *Malassezia* [64, 65]. To enhance the applicability of biofinishes in wood protection more studies are required on the availability of oil (components) at the wood surface and their role in fungal growth.

In addition, the water conditions on the surface of oil-treated wood samples that enables biofinish formation might favour growth of *Aureobasidium*. At first sight, the surfaces of wood samples treated with oils seem to be dry, unless it rains. However, dew drops have been noticed frequently on the hydrophobic surfaces during outdoor exposure. Although a few fungal species including *Aureobasidium* sp. and *Cladosporium* spp. are known to survive periods of low relative humidity [67, 68], fungal structures of *Aureobasidium* might benefit more than others from the considerably wet conditions. For example, this genus quickly formed visible colonies after inoculation of the biomass on MEA plates (water activity 0.99), while other genera needed more time to appear (data not shown). A detailed study on the water conditions on oil-treated wood samples and the impact of different water conditions on growth of wood-inhabiting genera should be performed.

## Conclusions

The presence of the common wood stain fungus *Aureobasidium* in biofinishes on oil-treated wood with both culturing- and DNA-based techniques is demonstrated. Moreover, the frequent predominance of *Aureobasidium* emphasises the importance of this genus as biofinish component. The importance of other genera, such as *Lapidomyces* and *Superstratomyces*, in biofinish formation has to be considered, but is not recognized for all other detected wood inhibiting fungi.

## Additional files

**Additional file 1: Table S1**. The total CFU concentration and the Aureobasidium CFU percentage of each wood sample (r. lins. oil = raw linseed oil, st. lins. oil = raw linseed oil, underlined numbers = estimated number, CFU count below 10).

**Additional file 2: Table S2.** Fungal isolates of each colony type with their culture collection numbers and GenBank accession numbers for the sequenced loci.

**Additional file 3: Table S3.** ITS specific clones inferred from biofinish DNA, their identification and GenBank accession number.

**Additional file 4: Table S4.** Illumina amplicon sequences identified by a BLAST search against the database of GenBank, Q-bank and CBS (type strains of the CBS Barcoding database were selected from data generated until September 2015). Sequences were identified to genus level when possible and marked as ‘unidentified genus’ when BLAST resulted in hits with an identity below 97%. The number of reads of each unique sequence is listed for each sample.
