# Supplementary material for: The fungal composition of natural biofinishes on oil-treated wood
Source: Fungal Biol Biotechnol. 2017 Jan 26;4:2. doi: 10.1186/s40694-017-0030-5 (PMC5611603; doi:10.1186/s40694-017-0030-5)
Supplement: Supplementary file 1 — Additional file 1: Table S1. The total CFU concentration and the Aureobasidium CFU percentage of each wood sample (r. lins. oil = raw linseed oil, st. lins. oil = raw linseed oil, underlined numbers = estimated number, CFU count below 10). [file 40694_2017_30_MOESM1_ESM.docx]

## Supplementary data – Table S1

Table S1: The total CFU concentration and the *Aureobasidium* CFU percentage of each wood sample (r. lins. oil = raw linseed oil, st. lins. oil = raw linseed oil, underlined numbers = estimated number , CFU count below 10)

| **Sample set** | **Wood species** | **Treatment** | **Biofinish present** | **Total CFU** | | ***Aureobasidium* CFU** | |
| --- | --- | --- | --- | --- | --- | --- | --- |
|  |  |  |  | Concentration | | percentage of total | |
|  |  |  |  | **MEA P/S** | **DG18** | **MEA P/S** | **DG18** |
|  |  |  |  | CFU/cm² | CFU/cm² | (%) | (%) |
| 1 (NL) | pine sw | r. lins. oil | yesᵃ | 3E+04 | 3E+04 | **8** | **6** |
|  |  |  |  | 1E+04 | 7E+03 | **20** | **44** |
|  |  |  |  | 2E+04 | 3E+04 | **9** | **7** |
|  |  | st. lins. oil | no | 4E+04 | 2E+04 | **0** | **1** |
|  |  |  |  | 4E+04 | 2E+04 | **2** | **4** |
|  |  |  |  | 5E+04 | 2E+04 | **3** | **7** |
|  |  | olive oil | yesᵃ | 6E+03 | 4E+03 | **2** | **3** |
|  |  |  |  | 5E+04 | 5E+04 | 1,0 | 0,3 |
|  |  |  |  | 2E+04 | 3E+04 | **9** | **9** |
|  |  | no oil | no | 6E+03 | 8E+03 | **13** | **7** |
|  |  |  |  | 1E+04 | 1E+04 | 2 | 1 |
|  |  |  |  | 1E+04 | 7E+03 | 3 | 4 |
|  | spruce | r. lins. oil | no | 4E+02 | 4E+02 | **66** | **81** |
|  |  |  |  | 1E+03 | 9E+02 | **98** | **95** |
|  |  |  |  | 2E+03 | 8E+02 | **47** | **88** |
|  |  | st. lins. oil | no | 1E+03 | 2E+02 | **5** | **39** |
|  |  |  |  | 9E+02 | 2E+02 | **24** | **59** |
|  |  |  |  | 6E+03 | 1E+03 | **10** | **77** |
|  |  | olive oil | yesᵃ | 2E+03 | 2E+03 | **29** | **23** |
|  |  |  |  | 8E+03 | 1E+04 | **9** | **0,5** |
|  |  |  |  | 3E+03 | 2E+03 | **41** | **48** |
|  |  | no oil | no | 2E+02 | 1E+02 | 18 | 28 |
|  |  |  |  | 4E+02 | 1E+02 | 1 | 8 |
|  |  |  |  | 1E+03 | 1E+02 | **5** | **43** |
|  | ilomba | r. lins. oil | no | 2E+03 | 3E+03 | **14** | **10** |
|  |  |  |  | 2E+03 | 2E+03 | 1 | 0,1 |
|  |  |  |  | 2E+04 | 1E+04 | 1 | **1** |
|  |  | st. lins. oil | no | 2E+03 | 8E+02 | 0 | 1 |
|  |  |  |  | 4E+03 | 2E+03 | 0,2 | 1 |
|  |  |  |  | 1E+03 | 6E+02 | 4 | 8 |
|  |  | olive oil | yesᵃ | 9E+03 | 1E+04 | 4 | 3 |
|  |  |  |  | 8E+03 | 7E+03 | 2 | 3 |
|  |  |  |  | 3E+04 | 4E+04 | 4 | 3 |
|  |  | no oil | no | 8E+01 | 2E+02 | 0 | 0 |
|  |  |  |  | 1E+02 | 1E+02 | 0 | 0 |
|  |  |  |  | 2E+02 | 3E+02 | 3 | 1 |
| 2 (NL) | pine sw | r. lins. oil | yesᵃ | 1E+04 | 7E+03 | 2 | 2 |
|  |  |  |  | 2E+03 | 2E+03 | 37 | 54 |
|  |  |  |  | 2E+03 | 1E+03 | 3 | 17 |
|  |  |  |  | 3E+04 | 2E+04 | 4,6 | 6,8 |
|  |  |  |  | 2E+03 | 2E+03 | 25 | 49 |
|  |  |  |  | 7E+03 | 3E+03 | 21 | 41 |
|  |  |  |  | 2E+04 | 1E+04 | 9 | 12 |
|  |  |  |  | 2E+05 | 2E+05 | 0,03 | 0,02 |
|  |  |  |  | 3E+04 | 4E+03 | 0,1 | 1 |
|  |  |  |  | 1E+04 | 4E+03 | 0,4 | 3 |
| 3 (NL) | spruce | r. lins. oil | no | 1E+05 | 1E+05 | 8 | 11 |
|  | ilomba | r. lins. oil | no | 3E+05 | 4E+05 | 1 | 1 |
|  | pine sw | r. lins. oil | yesᵇ | 2E+04 | 3E+04 | 36 | 38 |
|  | pine sw | olive oil | yesᵇ | 4E+05 | 3E+05 | 0,5 | 0,2 |
|  | pine sw | no oil | no | 1E+04 | 1E+04 | 5 | 6 |
|  | pine hw | r. lins. oil | yesᵇ | 2E+04 | 3E+04 | 96 | 97 |
| 4 (SA) | spruce | r. lins. oil | no | 6E+03 | 6E+03 | 89 | 95 |
|  | ilomba | r. lins. oil | no | 9E+03 | 6E+03 | 83 | 96 |
|  | pine sw | r. lins. oil | no | 2E+02 | 2E+02 | 72 | 87 |
|  | pine sw | olive oil | yesᵃ | 1E+03 | 1E+03 | - | - |
|  | pine sw | no oil | no | 1E+03 | 2E+03 | - | - |
|  | pine hw | r. lins. oil | no | 3E+01 | 2E+01 | 46 | 70 |
| 5 (AUS) | spruce | r. lins. oil | no | 6E+03 | 5E+03 | 78 | 81 |
|  | ilomba | r. lins. oil | no | 1E+04 | 6E+03 | 28 | 48 |
|  | pine sw | r. lins. oil | no | 3E+04 | 2E+04 | 56 | 91 |
|  | pine sw | olive oil | no | 7E+04 | 5E+03 | 3 | 26 |
|  | pine sw | no oil | no | 2E+03 | 2E+03 | 76 | 58 |
|  | pine hw | r. lins. oil | no | 5E+04 | 1E+03 | 2 | 71 |
| 6 (NW) | spruce | r. lins. oil | no | 1E+03 | 1E+03 | 18 | 14 |
|  | ilomba | r. lins. oil | no | 5E+03 | 4E+03 | 14 | 18 |
|  | pine sw | r. lins. oil | yesᵃ | 6E+02 | 7E+02 | 13 | 4 |
|  | pine sw | olive oil | no | 7E+02 | 9E+02 | 0,0 | 0,0 |
|  | pine sw | no oil | no | 1E+03 | 8E+02 | 7 | 9 |
|  | pine hw | r. lins. oil | yesᵃ | 1E+03 | 7E+02 | 25 | 25 |

^a^ Biofinish assessment by van Nieuwenhuijzen et al. (2015); ^b^ Determination based on stain coverage
