# Supplementary material for: The fungal composition of natural biofinishes on oil-treated wood
Source: Fungal Biol Biotechnol. 2017 Jan 26;4:2. doi: 10.1186/s40694-017-0030-5 (PMC5611603; doi:10.1186/s40694-017-0030-5)
Supplement: Supplementary file 2 — Additional file 2: Table S2. Fungal isolates of each colony type with their culture collection numbers and GenBank accession numbers for the sequenced loci. [file 40694_2017_30_MOESM2_ESM.docx]

Table S2 : Fungal isolates of each colony type with their culture collection numbers and GenBank accession numbers for the sequenced loci.

| Colony type | Sample set | Locality | Substrate | DTO no. | CBS no. | GenBank accession numbers | |
| --- | --- | --- | --- | --- | --- | --- | --- |
|  |  |  |  |  |  | ITS | *LSU* |
| *Aureobasidium* | set 1 | The Netherlands | pine sw treated with stand linseed oil | 277-B5 | - | KT693630 | - |
|  |  |  |  | 277-B6 | - | KT693631 | - |
|  |  |  |  | 277-B7 | - | KT693632 | - |
|  |  |  |  | 277-B8 | - | KT693633 | - |
|  |  |  |  | 277-B9 | - | KT693634 | - |
|  |  |  |  | 277-C1 | - | KT693635 | - |
|  |  |  |  | 277-C2 | - | KT693636 | - |
|  |  |  | ilomba treated with olive oil | 277-C3 | - | KT693637 | - |
|  |  |  |  | 277-C4 | - | KT693638 | - |
|  |  |  |  | 277-C5 | - | KT693639 | - |
|  |  |  |  | 277-C6 | - | KT693640 | - |
|  |  |  |  | 277-C7 | - | KT693641 | - |
|  |  |  |  | 277-G5 | - | KT693642 | - |
|  |  |  |  | 277-G6 | - | KT693643 | - |
|  |  |  | spruce treated with olive oil | 277-F4 | - | KT693644 | - |
|  |  |  |  | 277-F5 | - | KT693645 | - |
|  |  |  |  | 277-F6 | - | KT693646 | - |
|  |  |  |  | 277-F7 | - | KT693647 | - |
|  |  |  |  | 277-F8 | - | KT693648 | - |
|  |  |  |  | 277-F9 | - | KT693649 | - |
|  |  |  |  | 277-G1 | - | KT693650 | - |
|  |  |  |  | 277-G2 | - | KT693651 | - |
|  |  |  |  | 277-G3 | - | KT693652 | - |
|  |  |  |  | 277-G4 | - | KT693653 | - |
|  | set 2 | The Netherlands | pine sw treated with raw linseed oil | 305-C8 | - | KX147568 | - |
|  |  |  |  | 305-C9 | - | KX147569 | - |
|  | set 4 | South Africa | spruce treated with raw linseed oil | 285-D3 | - | KT693667 | - |
|  |  |  |  | 285-D4 | - | KT693668 | - |
|  |  |  |  | 285-D5 | - | KT693669 | - |
|  |  |  |  | 296-E8 | CBS 140250 | KT693670 | - |
|  |  |  | ilomba treated with raw linseed oil | 285-D6 | - | KT693671 | - |
|  |  |  |  | 285-D7 | CBS 140251 | KT693672 | - |
|  |  |  |  | 285-D8 | - | KT693673 | - |
|  |  |  |  | 285-D9 | - | KT693674 | - |
|  |  |  | pine sw treated with raw linseed oil | 285-E1 | CBS 140252 | KT693675 | - |
|  |  |  |  | 285-E2 | CBS 140253 | KT693676 | - |
|  |  |  |  | 285-E3 | - | KT693677 | - |
|  |  |  |  | 285-E4 | CBS 140254 | KT693678 | - |
|  |  |  |  | 296-F6 | - | KT693679 | - |
|  |  |  |  | 296-G3 | - | KT693680 | - |
|  |  |  |  | 296-G4 | - | KT693681 | - |
|  |  |  |  | 296-G5 | CBS 140255 | KT693682 | - |
|  |  |  |  | 296-G6 | CBS 140256 | KT693683 | - |
|  |  |  | pine sw treated with olive oil | 285-E5 | CBS 140257 | KT693684 | - |
|  |  |  |  | 285-E6 | CBS 140258 | KT693685 | - |
|  |  |  |  | 285-E7 | - | KT693686 | - |
|  |  |  |  | 285-E8 | - | KT693687 | - |
|  |  |  | untreated pine sw | 296-F7 | - | KT693688 | - |
|  |  |  |  | 296-F8 | - | KT693689 | - |
|  |  |  |  | 296-F9 | CBS 140259 | KT693690 | - |
|  |  |  |  | 296-G1 | CBS 140260 | KT693691 | - |
|  | set 5 | Australia | spruce treated with raw linseed oil | 301-G5 | - | KT693692 | - |
|  |  |  |  | 301-G6 | - | KT693693 | - |
|  |  |  | ilomba treated with raw linseed oil | 301-G9 | CBS 140261 | KT693694 | - |
|  |  |  |  | 301-H1 | - | KT693695 | - |
|  |  |  |  | 301-H2 | - | KT693696 | - |
|  |  |  |  | 301-H3 | - | KT693697 | - |
|  |  |  | pine sw treated with raw linseed oil | 300-I2 | CBS 140262 | KT693698 | - |
|  |  |  |  | 300-I3 | CBS 140263 | KT693699 | - |
|  |  |  |  | 300-I4 | - | KT693700 | - |
|  |  |  |  | 300-I5 | - | KT693701 | - |
|  |  |  |  | 301-F7 | CBS 140264 | KT693702 | - |
|  |  |  |  | 301-F8 | - | KT693703 | - |
|  |  |  |  | 301-F9 | CBS 140265 | KT693704 | - |
|  |  |  | pine sw treated with olive oil | 300-I8 | - | KT693705 | - |
|  |  |  |  | 300-I9 | - | KT693706 | - |
|  |  |  | untreated pine sw | 301-A6 | - | KT693707 | - |
|  |  |  |  | 301-A7 | - | KT693708 | - |
|  |  |  |  | 301-A8 | - | KT693709 | - |
|  |  |  |  | 301-F4 | CBS 140266 | KT693710 | - |
|  | set 6 | Norway | spruce treated with raw linseed oil | 302-E1 | CBS 140267 | KT693711 | - |
|  |  |  |  | 302-E2 | - | KT693712 | - |
|  |  |  |  | 302-E3 | - | KT693713 | - |
|  |  |  | ilomba treated with raw linseed oil | 302-E9 | - | KT693714 | - |
|  |  |  |  | 302-F1 | CBS 140268 | KT693715 | - |
|  |  |  |  | 302-F2 | CBS 140269 | KT693716 | - |
|  |  |  | pine sw treated with raw linseed oil | 302-F7 | - | KT693717 | - |
|  |  |  |  | 302-F8 | - | KT693718 | - |
|  |  |  |  | 302-H8 | - | KT693719 | - |
|  |  |  |  | 302-H9 | - | KT693720 | - |
|  |  |  |  | 302-I1 | - | KT693721 | - |
|  |  |  |  | 302-I2 | - | KT693722 | - |
|  |  |  | pine sw treated with olive oil | 302-G3 | - | KT693723 | - |
|  |  |  | untreated pine sw | 302-H1 | - | KT693724 | - |
|  |  |  |  | 302-H2 | - | KT693725 | - |
|  |  |  |  | 302-H3 | - | KT693726 | - |
| Black yeasts | set 1 | The Netherlands | pine sw treated with raw linseed oil | 277-H2 | - | KX147570 | - |
|  |  |  | pine sw treated with stand linseed oil | 277-H3 | - | KX147571 | - |
|  |  |  |  | 277-H4 | - | KX147572 | - |
|  |  |  | untreated pine sw | 277-H9 | - | KX147573 | - |
|  |  |  | spruce treated with raw linseed oil | 277-G8 | - | KX147574 | - |
|  |  |  | spruce treated with stand linseed oil | 277-G9 | - | KX147575 | - |
|  |  |  |  | 277-H1 | - | KX147576 | - |
|  | set 2 | The Netherlands | pine sw treated with raw linseed oil | 305-D1 | - | KX147577 | KX171351 |
|  |  |  |  | 305-D2 | - | KX147578 | KX171352 |
|  |  |  |  | 305-D3 | - | KX147579 | - |
|  |  |  |  | 305-D4 | - | KX147580 | - |
|  | set 4 | South Africa | pine sw treated with raw linseed oil | 296-F4 | - | KX147581 | - |
|  |  |  |  | 296-F5 | - | KX147582 | - |
|  |  |  | spruce treated with raw linseed oil | 296-E9 | - | KX147583 | KX171353 |
|  |  |  |  | 296-F1 | - | KX147584 | KX171354 |
|  | set 5 | Australia | pine sw treated with olive oil | 301-A4 | - | KX147585 | - |
|  |  |  |  | 301-A5 | - | KX147586 | KX171355 |
|  |  |  | ilomba treated with raw linseed oil | 301-H4 | - | KX147587 | - |
|  |  |  |  | 301-H5 | - | KX147588 | KX171356 |
|  |  |  |  | 301-H6 | - | KX147589 | - |
|  |  |  |  | 301-H7 | - | KX147590 | KX171357 |
|  | set 6 | Norway | pine sw treated with raw linseed oil | 302-I7 | - | KX147591 | - |
|  |  |  |  | 302-I8 | - | KX147592 | - |
|  |  |  | ilomba treated with raw linseed oil | 302-F5 | - | KX147593 | KX171358 |
| *Cladosporium* | set 1 | The Netherlands | untreated ilomba | 278-A6 | - | KX147594 | - |
|  |  |  |  | 278-A7 | - | KX147595 | - |
|  |  |  |  | 278-A8 | - | KX147596 | - |
|  |  |  |  | 278-A9 | - | KX147597 | - |
| *Cryptococcus* | set 5 | Australia | pine sw treated with olive oil | 301-A1 | - | KX147598 | - |
|  |  |  |  | 301-A3 | - | KX147599 | - |
| *Didymellaceae* | set 1 | The Netherlands | ilomba treated with raw linseed oil | 278-A4 | - | KX147600 | KX171359 |
|  |  |  |  | 278-A5 | - | KX147601 | - |
| *Phacidiella* | set 2 | The Netherlands | pine sw treated with raw linseed oil | 305-C4 | - | KX147602 | - |
|  |  |  |  | 305-C5 | - | KX147603 | - |
| *Pleurophoma* | set 1 | The Netherlands | untreated ilomba | 277-D8 | - | KX147604 | KX171360 |
|  |  |  |  | 277-D9 | - | KX147605 | - |
| *Pyrenochaeta* | set 2 | The Netherlands | pine sw treated with raw linseed oil | 305-C6 | - | KX147606 | KX171361 |
|  |  |  |  | 305-C7 | - | KX147607 | - |
| *Superstratomyces* | set 1 | The Netherlands | pine sw treated with raw linseed oil | 277-H6 | CBS 140277 | KX950419 | KX950446 |
|  |  |  |  | 277-H7 | CBS 140278 | KX950420 | KX950447 |
|  |  |  |  | 277-I3 | CBS 140271 | KX950413 | KX950440 |
|  |  |  | pine sw treated with stand linseed oil | 277-C8 | CBS 140273 | KX950415 | KX950442 |
|  |  |  |  | 277-C9 | CBS 140274 | KX950416 | KX950443 |
|  |  |  |  | 277-H8 | CBS 140279 | KX950421 | KX950448 |
|  |  |  |  | 277-I4 | CBS 140281 | KX950423 | KX950450 |
|  |  |  |  | 277-I8 | CBS 140285 | KX950427 | KX950454 |
|  |  |  | spruce treated with olive oil | 277-I2 | CBS 140280 | KX950422 | KX950449 |
|  |  |  |  | 277-I6 | CBS 140283 | KX950425 | KX950452 |
|  |  |  |  | 277-I7 | CBS 140284 | KX950426 | KX950453 |
|  |  |  |  | 277-I9 | CBS 140286 | KX950428 | KX950455 |
|  |  |  |  | 278-A3 | CBS 140287 | KX950430 | KX950457 |
|  |  |  | ilomba treated with raw linseed oil | 277-I5 | CBS 140282 | KX950424 | KX950451 |
|  |  |  |  | 278-A2 | CBS 140343 | KX950429 | KX950456 |
|  |  |  | ilomba treated with olive oil | 277-D2 | CBS 140270 | KX950412 | KX950439 |
|  |  |  |  | 277-D3 | CBS 140275 | KX950417 | KX950444 |
|  |  |  |  | 277-D4 | CBS 140276 | KX950418 | KX950445 |
|  | set 2 | The Netherlands | pine sw treated with raw linseed oil | 305-D9 | CBS 140288 | KX950431 | KX950458 |
|  |  |  |  | 305-E1 | CBS 140272 | KX950414 | KX950441 |
|  |  |  |  | 305-E2 | CBS 140289 | KX950432 | KX950459 |
|  |  |  |  | 305-E3 | CBS 140344 | KX950433 | KX950460 |
| *Cyanodermella* | set 5 (Aus) | set 5 (Aus) | pine sw treated with raw linseed oil | 301-G1 | CBS 140290 | KX950434 | KX950461 |
|  |  |  |  | 301-G2 | - | KX950435 | - |
|  |  |  |  | 301-G3 | - | KX950436 | - |
|  |  |  |  | 301-G4 | - | KX950437 | - |
| *Sydowia* | set 6 | Norway | pine sw treated with raw linseed oil | 302-F9 | - | KX147608 | - |
|  |  |  |  | 302-G1 | - | KX147609 | - |
|  |  |  |  | 302-G2 | - | KX147610 | - |
|  |  |  |  | 302-G7 | - | KX147611 | - |
|  |  |  |  | 302-G8 | - | KX147612 | - |
|  |  |  |  | 302-G9 | - | KX147613 | - |
|  |  |  | pine sw treated with olive oil | 302-G4 | - | KX147614 | - |
|  |  |  |  | 302-G5 | - | KX147615 | - |
|  |  |  |  | 302-G6 | - | KX147616 | - |
|  |  |  | untreated pine sw | 302-H4 | - | KX147617 | - |
|  |  |  |  | 302-H5 | - | KX147618 | - |
|  |  |  |  | 302-H6 | - | KX147619 | - |
|  |  |  |  | 302-H7 | - | KX147620 | - |
|  |  |  | spruce treated with raw linseed oil | 302-E4 | - | KX147621 | - |
|  |  |  |  | 302-E5 | - | KX147622 | - |
|  |  |  |  | 302-E6 | - | KX147623 | - |
|  |  |  |  | 302-E7 | - | KX147624 | - |
|  |  |  |  | 302-E8 | - | KX147625 | - |
|  |  |  | ilomba treated with raw linseed oil | 302-F3 | - | KX147626 | - |
|  |  |  |  | 302-F4 | - | KX147627 | - |
| *Taphrina* | set 1 | The Netherlands | pine sw treated with raw linseed oil | 277-I1 | - | KX147628 | - |
|  | set 2 | The Netherlands | pine sw treated with raw linseed oil | 305-D5 | - | KX147629 | - |
|  |  |  |  | 305-D6 | - | KX147630 | - |
|  |  |  |  | 305-D7 | - | KX147631 | - |
|  |  |  |  | 305-D8 | - | KX147632 | - |
