# Supplementary material for: The fungal composition of natural biofinishes on oil-treated wood
Source: Fungal Biol Biotechnol. 2017 Jan 26;4:2. doi: 10.1186/s40694-017-0030-5 (PMC5611603; doi:10.1186/s40694-017-0030-5)
Supplement: Supplementary file 3 — Additional file 3: Table S3. ITS specific clones inferred from biofinish DNA, their identification and GenBank accession number. [file 40694_2017_30_MOESM3_ESM.docx]

Table S3: ITS specific clones inferred from biofinish DNA, their identification and GenBank accession number

| Clone | Genus identification | Additional info on best GenBank hits | GenBank accession no. |
| --- | --- | --- | --- |
|  |  |  |  |
| PRL.1.2 | *Phaeococcomyces* | - | KX147766 |
| PRL.1.17 | *Lapidomyces* | - | KX147767 |
| PRL.1.18 | *Lapidomyces* | - | KX147768 |
| PRL.1.19 | unidentified | - | KX147769 |
| PRL.1.23 | *Lapidomyces* | - | KX147770 |
| PRL.1.28 | unidentified | *Sarcinomyces* / Rizosphaera, query 100%, ident. 82% | KX147771 |
| PRL.1.29 | unidentified | - | KX147772 |
| PRL.1.35 | unidentified | *Sarcinomyces* / Rizosphaera, query 100%, ident. 82% | KX147773 |
| PRL.1.36 | *Lapidomyces* | - | KX147774 |
| PRL.1.37 | unidentified | *Sarcinomyces* / *Rizosphaera*, query 100%, ident. 82% | KX147775 |
| PRL.1.40 | *Lapidomyces* | - | KX147776 |
| PRL.1.49 | *Lapidomyces* | - | KX147777 |
| PRL.1.52 | *Lapidomyces* | - | KX147778 |
| PRL.1.55 | *Lapidomyces* | - | KX147779 |
| PRL.1.56 | *Lapidomyces* | - | KX147780 |
| PRL.1.57 | *Epicoccum* | - | KX147781 |
| PRL.1.59 | unidentified | *Septoria*, query 99%, identity 80% | KX147782 |
| PRL.1.60 | *Lapidomyces* | - | KX147783 |
| PRL.1.64 | *Lapidomyces* | - | KX147784 |
| PRL.1.65 | *Lapidomyces* | - | KX147785 |
| PRL.1.66 | unidentified | *Sporobolomyces*, query 100%, identity 85% | KX147786 |
| PRL.1.72 | unidentified | *Septoria*, query 100%, identity 80% | KX147787 |
| PRL.1.73 | unidentified | *Sarcinomyces* / *Rizosphaera*, query 100%, ident. 82% | KX147788 |
| PRL.1.83 | *Lapidomyces* | - | KX147789 |
| PRL.1.84 | *Lapidomyces* | - | KX147790 |
| PRL.1.87 | unidentified | *Sarcinomyces* / *Rizosphaera*, query 100%, ident. 82% | KX147791 |
| PRL.1.05 | *Aureobasidium* | - | KT693388 |
| PRL.1.06 | *Aureobasidium* | - | KT693389 |
| PRL.1.09 | *Aureobasidium* | - | KU671015 |
| PRL.1.21 | *Aureobasidium* | - | KT693390 |
| PRL.1.25 | *Aureobasidium* | - | KT693391 |
| PRL.1.26 | *Aureobasidium* | - | KT693392 |
| PRL.1.27 | *Aureobasidium* | - | KT693393 |
| PRL.1.30 | *Aureobasidium* | - | KT693394 |
| PRL.1.31 | *Aureobasidium* | - | KT693395 |
| PRL.1.32 | *Aureobasidium* | - | KU671016 |
| PRL.1.33 | *Aureobasidium* | - | KT693396 |
| PRL.1.34 | *Aureobasidium* | - | KT693397 |
| PRL.1.38 | *Aureobasidium* | - | KU671017 |
| PRL.1.39 | *Aureobasidium* | - | KT693398 |
| PRL.1.50 | *Aureobasidium* | - | KT693399 |
| PRL.1.53 | *Aureobasidium* | - | KT693401 |
| PRL.1.54 | *Aureobasidium* | - | KT693402 |
| PRL.1.58 | *Aureobasidium* | - | KT693403 |
| PRL.1.61 | *Aureobasidium* | - | KT693404 |
| PRL.1.62 | *Aureobasidium* | - | KT693405 |
| PRL.1.67 | *Aureobasidium* | - | KT693406 |
| PRL.1.69 | *Aureobasidium* | - | KT693407 |
| PRL.1.70 | *Aureobasidium* | - | KT693408 |
| PRL.1.71 | *Aureobasidium* | - | KT693409 |
| PRL.1.74 | *Aureobasidium* | - | KT693410 |
| PRL.1.75 | *Aureobasidium* | - | KT693411 |
| PRL.1.76 | *Aureobasidium* | - | KT693412 |
| PRL.1.77 | *Aureobasidium* | - | KT693413 |
| PRL.1.78 | *Aureobasidium* | - | KT693414 |
| PRL.1.79 | *Aureobasidium* | - | KT693415 |
| PRL.1.81 | *Aureobasidium* | - | KT693416 |
| PRL.1.82 | *Aureobasidium* | - | KT693417 |
| PRL.1.85 | *Aureobasidium* | - | KT693418 |
| PRL.1.86 | *Aureobasidium* | - | KT693419 |
| PRL.1.88 | *Aureobasidium* | - | KT693420 |
| PRL.2.1 | *Lapidomyces* | - | KX147792 |
| PRL.2.4 | unidentified | *Sarcinomyces* / *Rizosphaera*, query 100%, ident. 82% | KX147793 |
| PRL.2.6 | unidentified | *Sarcinomyces* / *Rizosphaera*, query 100%, ident. 82% | KX147794 |
| PRL.2.21 | unidentified | *Phaeococcomyces*, query 99%, identity 92% | KX147795 |
| PRL.2.24 | *Lapidomyces* | - | KX147796 |
| PRL.2.28 | *Lapidomyces* | - | KX147797 |
| PRL.2.30 | *Lapidomyces* | - | KX147798 |
| PRL.2.31 | *Lapidomyces* | - | KX147799 |
| PRL.2.32 | *Lapidomyces* | - | KX147800 |
| PRL.2.33 | *Lapidomyces* | - | KX147801 |
| PRL.2.40 | unidentified | *Septorioides*, query 100%, identity 80% | KX147802 |
| PRL.2.41 | unidentified | *Sarcinomyces* / *Rizosphaera*, query 100%, ident. 82% | KX147803 |
| PRL.2.42 | unidentified | *Sarcinomyces* / *Rizosphaera*, query 100%, ident. 82% | KX147804 |
| PRL.2.45 | *Lapidomyces* | - | KX147805 |
| PRL.2.46 | unidentified | *Sarcinomyces* / *Rizosphaera*, query 100%, ident. 82% | KX147806 |
| PRL.2.48 | unidentified | *Septorioides*, query 100%, identity 80% | KX147807 |
| PRL.2.52 | *Lapidomyces* | - | KX147808 |
| PRL.2.53 | unidentified | *Septorioides*, query 100%, identity 80% | KX147809 |
| PRL.2.54 | *Thaprina* | - | KX147810 |
| PRL.2.55 | unidentified | *Lapidomyces*, query 67%, identity 96% | KX147811 |
| PRL.2.56 | unidentified | *Sarcinomyces* / *Rizosphaera*, query 100%, ident. 82% | KX147812 |
| PRL.2.59 | *Lapidomyces* | - | KX147813 |
| PRL.2.60 | unidentified | *Diplodia*, query 78%, identity 88% | KX147814 |
| PRL.2.61 | *Thaprina* | query 68%, identity 100% | KX147567* |
| PRL.2.66 | *Thaprina* | - | KX147815 |
| PRL.2.67 | *Lapidomyces* | - | KX147816 |
| PRL.2.68 | unidentified | *Sarcinomyces* / *Rizosphaera*, query 100%, ident. 82% | KX147817 |
| PRL.2.69 | *Lapidomyces* | - | KX147818 |
| PRL.2.71 | *Exophiala* | - | KX147819 |
| PRL.2.72 | unidentified | *Sarcinomyces* / *Rizosphaera*, query 100%, ident. 82% | KX147820 |
| PRL.2.79 | *Lapidomyces* | - | KX147821 |
| PRL.2.84 | unidentified | *Sarcinomyces* / *Rizosphaera*, query 100%, ident. 82% | KX147822 |
| PRL.2.85 | *Lapidomyces* | - | KX147823 |
| PRL.2.02 | *Aureobasidium* | - | KT693421 |
| PRL.2.03 | *Aureobasidium* | - | KT693422 |
| PRL.2.05 | *Aureobasidium* | - | KT693423 |
| PRL.2.07 | *Aureobasidium* | - | KT693424 |
| PRL.2.08 | *Aureobasidium* | - | KT693425 |
| PRL.2.22 | *Aureobasidium* | - | KT693426 |
| PRL.2.23 | *Aureobasidium* | - | KT693427 |
| PRL.2.25 | *Aureobasidium* | - | KT693428 |
| PRL.2.26 | *Aureobasidium* | - | KT693429 |
| PRL.2.27 | *Aureobasidium* | - | KT693430 |
| PRL.2.29 | *Aureobasidium* | - | KT693431 |
| PRL.2.34 | *Aureobasidium* | - | KT693432 |
| PRL.2.35 | *Aureobasidium* | - | KT693433 |
| PRL.2.36 | *Aureobasidium* | - | KT693434 |
| PRL.2.37 | *Aureobasidium* | - | KT693435 |
| PRL.2.38 | *Aureobasidium* | - | KT693436 |
| PRL.2.39 | *Aureobasidium* | - | KT693437 |
| PRL.2.43 | *Aureobasidium* | - | KT693438 |
| PRL.2.44 | *Aureobasidium* | - | KT693439 |
| PRL.2.47 | *Aureobasidium* | - | KT693440 |
| PRL.2.49 | *Aureobasidium* | - | KT693441 |
| PRL.2.50 | *Aureobasidium* | - | KT693442 |
| PRL.2.51 | *Aureobasidium* | - | KT693443 |
| PRL.2.62 | *Aureobasidium* | - | KT693444 |
| PRL.2.63 | *Aureobasidium* | - | KT693445 |
| PRL.2.64 | *Aureobasidium* | - | KT693446 |
| PRL.2.65 | *Aureobasidium* | - | KT693447 |
| PRL.2.70 | *Aureobasidium* | - | KT693448 |
| PRL.2.73 | *Aureobasidium* | - | KT693449 |
| PRL.2.74 | *Aureobasidium* | - | KU671018 |
| PRL.2.76 | *Aureobasidium* | - | KT693450 |
| PRL.2.78 | *Aureobasidium* | - | KT693451 |
| PRL.2.80 | *Aureobasidium* | - | KT693452 |
| PRL.2.82 | *Aureobasidium* | - | KT693453 |
| PRL.2.83 | *Aureobasidium* | - | KT693454 |
| PRL.2.86 | *Aureobasidium* | - | KT693455 |
| PRL.2.87 | *Aureobasidium* | - | KT693456 |
| PRL.3.3 | *Thaprina* | - | KX147824 |
| PRL.3.4 | unidentified | *Sarcinomyces* / *Rizosphaera*, query 100%, ident. 82% | KX147825 |
| PRL.3.7 | unidentified | *Sarcinomyces* / *Rizosphaera*, query 100%, ident. 82% | KX147826 |
| PRL.3.10 | *Exophiala* | - | KX147827 |
| PRL.3.13 | *Phaeococcomyces* | - | KX147828 |
| PRL.3.14 | unidentified | *Diplodia*, query 64%, identity 87% | KX147829 |
| PRL.3.26 | *Phaeococcomyces* | - | KX147830 |
| PRL.3.34 | *Phaeococcomyces* | - | KX147831 |
| PRL.3.46 | *Phaeococcomyces* | - | KX147832 |
| PRL.3.48 | *Lapidomyces* | - | KX147833 |
| PRL.3.49 | *Exophiala* | - | KX147834 |
| PRL.3.50 | unidentified | *Kellermania* query 66%, identity 87% | KX147835 |
| PRL.3.52 | *Lapidomyces* | - | KX147836 |
| PRL.3.58 | unidentified | *Sarcinomyces* / *Rizosphaera*, query 100%, ident. 82% | KX147837 |
| PRL.3.59 | unidentified | *Sarcinomyces* / *Rizosphaera*, query 100%, ident. 82% | KX147838 |
| PRL.3.62 | *Phaeococcomyces* | - | KX147839 |
| PRL.3.63 | unidentified | *Phaeococcomyces*, query 99%, identity 96% | KX147840 |
| PRL.3.64 | *Lapidomyces* | - | KX147841 |
| PRL.3.69 | *Exophiala* | - | KX147842 |
| PRL.3.72 | *Lapidomyces* | - | KX147843 |
| PRL.3.75 | *Exophiala* | - | KX147844 |
| PRL.3.76 | *Exophiala* | - | KX147845 |
| PRL.3.77 | *Thaprina* | - | KX147846 |
| PRL.3.79 | *Exophiala* | - | KX147847 |
| PRL.3.02 | *Aureobasidium* | - | KT693457 |
| PRL.3.06 | *Aureobasidium* | - | KT693458 |
| PRL.3.08 | *Aureobasidium* | - | KT693459 |
| PRL.3.09 | *Aureobasidium* | - | KT693460 |
| PRL.3.16 | *Aureobasidium* | - | KU671021* |
| PRL.3.17 | *Aureobasidium* | - | KT693461 |
| PRL.3.19 | *Aureobasidium* | - | KU671022* |
| PRL.3.21 | *Aureobasidium* | - | KT693462 |
| PRL.3.22 | *Aureobasidium* | - | KT693463 |
| PRL.3.23 | *Aureobasidium* | - | KT693464 |
| PRL.3.24 | *Aureobasidium* | - | KT693465 |
| PRL.3.25 | *Aureobasidium* | - | KT693466 |
| PRL.3.28 | *Aureobasidium* | - | KT693467 |
| PRL.3.31 | *Aureobasidium* | - | KT693468 |
| PRL.3.32 | *Aureobasidium* | - | KT693469 |
| PRL.3.36 | *Aureobasidium* | - | KT693470 |
| PRL.3.37 | *Aureobasidium* | - | KT693471 |
| PRL.3.39 | *Aureobasidium* | - | KT693472 |
| PRL.3.42 | *Aureobasidium* | - | KT693473 |
| PRL.3.43 | *Aureobasidium* | - | KT693474 |
| PRL.3.44 | *Aureobasidium* | - | KT693475 |
| PRL.3.47 | *Aureobasidium* | - | KT693476 |
| PRL.3.51 | *Aureobasidium* | - | KT693477 |
| PRL.3.54 | *Aureobasidium* | - | KT693478 |
| PRL.3.55 | *Aureobasidium* | - | KT693479 |
| PRL.3.56 | *Aureobasidium* | - | KT693480 |
| PRL.3.57 | *Aureobasidium* | - | KT693481 |
| PRL.3.65 | *Aureobasidium* | - | KT693482 |
| PRL.3.66 | *Aureobasidium* | - | KT693483 |
| PRL.3.68 | *Aureobasidium* | - | KU671019 |
| PRL.3.70 | *Aureobasidium* | - | KU671023 |
| PRL.3.71 | *Aureobasidium* | - | KU671020* |
| PRL.3.73 | *Aureobasidium* | - | KT693484 |
| PRL.3.74 | *Aureobasidium* | - | KT693485 |
| PRL.3.78 | *Aureobasidium* | - | KT693486 |
| PRL.3.80 | *Aureobasidium* | - | KT693487 |
| PRL.3.82 | *Aureobasidium* | - | KT693488 |
| PRL.3.83 | *Aureobasidium* | - | KT693489 |
| PO.1.1 | unidentified | *Phaeococcomyces*, query 100%, identity 95% | KX147848 |
| PO.1.2 | unidentified | *Exophiala*, query 100%, identity 96% | KX147849 |
| PO.1.3 | unidentified | *Sarcinomyces* / *Rizosphaera*, query 100%, ident. 82% | KX147850 |
| PO.1.5 | unidentified | *Sarcinomyces* / *Rizosphaera*, query 100%, ident. 82% | KX147851 |
| PO.1.6 | unidentified | *Sarcinomyces* / *Rizosphaera*, query 100%, ident. 82% | KX147852 |
| PO.1.7 | *Exophiala* | - | KX147853 |
| PO.1.11 | unidentified | *Sarcinomyces* / *Rizosphaera*, query 100%, ident. 82% | KX147854 |
| PO.1.12 | unidentified | *Septorioides*, query 100%, identity 81% | KX147855 |
| PO.1.14 | *Cladosporium* | - | KX147856 |
| PO.1.16 | *Septorioides* | *Septorioides*, query 100%, identity 81% | KX147857 |
| PO.1.17 | unidentified | *Lapidomyces*, query 64%, ident. 95% | KX148026* |
| PO.1.18 | unidentified | *Sarcinomyces* / *Rizosphaera*, query 100%, ident. 82% | KX147858 |
| PO.1.22 | unidentified | *Sarcinomyces* / *Rizosphaera*, query 100%, ident. 82% | KX147859 |
| PO.1.24 | unidentified | *Sarcinomyces* / *Rizosphaera*, query 100%, ident. 82% | KX147860 |
| PO.1.25 | unidentified | *Sarcinomyces* / *Rizosphaera*, query 100%, ident. 82% | KX147861 |
| PO.1.26 | *Capronia* | - | KX147862 |
| PO.1.27 | unidentified | *Sarcinomyces* / *Rizosphaera*, query 100%, ident. 82% | KX147863 |
| PO.1.28 | unidentified | *Sarcinomyces* / *Rizosphaera*, query 100%, ident. 82% | KX147864 |
| PO.1.29 | unidentified | *Sarcinomyces* / *Rizosphaera*, query 100%, ident. 82% | KX147865 |
| PO.1.31 | *Exophiala* | - | KX147866 |
| PO.1.33 | unidentified | *Sarcinomyces* / *Rizosphaera*, query 100%, ident. 82% | KX147867 |
| PO.1.35 | unidentified | *Sarcinomyces* / *Rizosphaera*, query 100%, ident. 82% | KX147868 |
| PO.1.36 | unidentified | *Sarcinomyces* / *Rizosphaera*, query 100%, ident. 82% | KX147869 |
| PO.1.38 | *Exophiala* | - | KX147870 |
| PO.1.39 | *Lapidomyces* | - | KX147871 |
| PO.1.40 | unidentified | *Septorioides*, query 100%, identity 81% | KX147872 |
| PO.1.41 | unidentified | *Phaeococcomyces*, query 96%, identity 94% | KX147873 |
| PO.1.42 | *Lapidomyces* | - | KX147874 |
| PO.1.43 | unidentified | *Sarcinomyces* / *Rizosphaera*, query 100%, ident. 82% | KX147875 |
| PO.1.44 | *Lapidomyces* | - | KX147876 |
| PO.1.47 | *Lapidomyces* | - | KX147877 |
| PO.1.48 | unidentified | *Sarcinomyces* / *Rizosphaera*, query 100%, ident. 82% | KX147878 |
| PO.1.49 | *Lapidomyces* | - | KX147879 |
| PO.1.51 | *Lapidomyces* | - | KX147880 |
| PO.1.52 | *Lapidomyces* | - | KX147881 |
| PO.1.53 | unidentified | *Sarcinomyces* / *Rizosphaera*, query 100%, ident. 82% | KX147882 |
| PO.1.54 | unidentified | *Sarcinomyces* / *Rizosphaera*, query 100%, ident. 82% | KX147883 |
| PO.1.55 | *Lapidomyces* | - | KX147884 |
| PO.1.58 | *Lapidomyces* | - | KX147885 |
| PO.1.60 | unidentified | *Sarcinomyces* / *Rizosphaera*, query 100%, ident. 82% | KX147886 |
| PO.1.61 | unidentified | (*Neo*)*catenulostroma*, query 100% , ident. 95% | KX147887 |
| PO.1.62 | unidentified | (*Neo*)*catenulostroma*, query 100% , ident. 94% | KX147888 |
| PO.1.64 | unidentified | *Sarcinomyces* / *Rizosphaera*, query 100%, ident. 82% | KX147889 |
| PO.1.65 | *Lapidomyces* | - | KX147890 |
| PO.1.66 | unidentified | *Sarcinomyces* / *Rizosphaera*, query 100%, ident. 82% | KX147891 |
| PO.1.67 | *Lapidomyces* | - | KX147892 |
| PO.1.69 | *Capronia* | - | KX147893 |
| PO.1.70 | *Lapidomyces* | - | KX147894 |
| PO.1.71 | *Lapidomyces* | - | KX147895 |
| PO.1.72 | *Lapidomyces* | - | KX147896 |
| PO.1.74 | *Lapidomyces* | - | KX147897 |
| PO.1.76 | unidentified | *Sarcinomyces* / *Rizosphaera*, query 100%, ident. 82% | KX147898 |
| PO.1.77 | unidentified | *Sarcinomyces* / *Rizosphaera*, query 100%, ident. 82% | KX147899 |
| PO.1.78 | *Lapidomyces* | - | KX147900 |
| PO.1.79 | unidentified | *Sarcinomyces* / *Rizosphaera*, query 100%, ident. 82% | KX147901 |
| PO.1.80 | unidentified | *Sarcinomyces* / *Rizosphaera*, query 100%, ident. 82% | KX147902 |
| PO.1.82 | unidentified | *Sarcinomyces* / *Rizosphaera*, query 100%, ident. 82% | KX147903 |
| PO.1.83 | *Lapidomyces* | - | KX147904 |
| PO.1.84 | unidentified | *Sarcinomyces* / *Rizosphaera*, query 100%, ident. 82% | KX147905 |
| PO.1.86 | unidentified | *Sarcinomyces* / *Rizosphaera*, query 100%, ident. 82% | KX147906 |
| PO.1.87 | *Lapidomyces* | - | KX147907 |
| PO.1.88 | *Lapidomyces* | - | KX147908 |
| PO.1.13 | *Aureobasidium* | - | KT693490 |
| PO.1.37 | *Aureobasidium* | - | KT693491 |
| PO.1.45 | *Aureobasidium* | - | KT693492 |
| PO.1.50 | *Aureobasidium* | - | KT693493 |
| PO.1.59 | *Aureobasidium* | - | KT693494 |
| PO.1.68 | *Aureobasidium* | - | KT693495 |
| PO.1.73 | *Aureobasidium* | - | KT693496 |
| PO.1.75 | *Aureobasidium* | - | KU671024* |
| PO.1.81 | *Aureobasidium* | - | KT693497 |
| PO.2.2 | unidentified | *Penidiella*, query 28%, identity 84% | KX147909 |
| PO.2.3 | unidentified | *Sarcinomyces* / *Rizosphaera*, query 100%, ident. 82% | KX147910 |
| PO.2.4 | unidentified | *Sarcinomyces* / *Rizosphaera*, query 100%, ident. 82% | KX147911 |
| PO.2.5 | unidentified | Penidiella, query 28%, identity 84% | KX147912 |
| PO.2.6 | unidentified | *Sarcinomyces* / *Rizosphaera*, query 100%, ident. 82% | KX147913 |
| PO.2.11 | unidentified | *Sarcinomyces* / *Rizosphaera*, query 100%, ident. 82% | KX147914 |
| PO.2.12 | unidentified | *Sarcinomyces* / *Rizosphaera*, query 100%, ident. 82% | KX147915 |
| PO.2.13 | unidentified | *Septorioides*, query 100%, identity 80% | KX147916 |
| PO.2.14 | unidentified | *Sarcinomyces* / *Rizosphaera*, query 100%, ident. 82% | KX147917 |
| PO.2.15 | unidentified | *Sarcinomyces* / *Rizosphaera*, query 100%, ident. 82% | KX147918 |
| PO.2.16 | unidentified | *Septorioides*, query 100%, identity 80% | KX147919 |
| PO.2.25 | unidentified | *Sarcinomyces* / *Rizosphaera*, query 100%, ident. 82% | KX147920 |
| PO.2.26 | *Lapidomyces* | - | KX147921 |
| PO.2.27 | *Lapidomyces* | - | KX147922 |
| PO.2.28 | unidentified | *Sarcinomyces* / *Rizosphaera*, query 100%, ident. 82% | KX147923 |
| PO.2.29 | unidentified | *Sarcinomyces* / *Rizosphaera*, query 100%, ident. 82% | KX147924 |
| PO.2.30 | *Exophiala* | - | KX147925 |
| PO.2.31 | unidentified | (*Neo*)*catenulostroma*, query 100%, ident. 94% | KX147926 |
| PO.2.35 | unidentified | *Sarcinomyces* / *Rizosphaera*, query 100%, ident. 82% | KX147927 |
| PO.2.36 | unidentified | *Sarcinomyces* / *Rizosphaera*, query 100%, ident. 82% | KX147928 |
| PO.2.37 | unidentified | *Sarcinomyces* / *Rizosphaera*, query 100%, ident. 82% | KX147929 |
| PO.2.38 | unidentified | *Sarcinomyces* / *Rizosphaera*, query 100%, ident. 82% | KX147930 |
| PO.2.39 | unidentified | *Penidiella*, query 28%, identity 84% | KX147931 |
| PO.2.42 | unidentified | *Sarcinomyces* / *Rizosphaera*, query 100%, ident. 82% | KX147932 |
| PO.2.44 | unidentified | *Sarcinomyces* / *Rizosphaera*, query 100%, ident. 82% | KX147933 |
| PO.2.45 | unidentified | *Sarcinomyces* / *Rizosphaera*, query 100%, ident. 82% | KX147934 |
| PO.2.46 | unidentified | *Sarcinomyces* / *Rizosphaera*, query 100%, ident. 82% | KX147935 |
| PO.2.47 | unidentified | *Sarcinomyces* / *Rizosphaera*, query 100%, ident. 82% | KX147936 |
| PO.2.50 | unidentified | *Sarcinomyces* / *Rizosphaera*, query 100%, ident. 82% | KX147937 |
| PO.2.51 | unidentified | *Sarcinomyces* / *Rizosphaera*, query 100%, ident. 82% | KX147938 |
| PO.2.52 | *Lapidomyces* | - | KX147939 |
| PO.2.53 | unidentified | *Sarcinomyces* / *Rizosphaera*, query 100%, ident. 82% | KX147940 |
| PO.2.54 | unidentified | *Endosporium* , query 95%, identity 81% | KX147941 |
| PO.2.55 | unidentified | *Sarcinomyces* / *Rizosphaera*, query 100%, ident. 82% | KX147942 |
| PO.2.57 | unidentified | *Sarcinomyces* / *Rizosphaera*, query 100%, ident. 82% | KX147943 |
| PO.2.58 | *Lapidomyces* | - | KX147944 |
| PO.2.59 | unidentified | *Sarcinomyces* / *Rizosphaera*, query 100%, ident. 82% | KX147945 |
| PO.2.60 | unidentified | *Sarcinomyces* / *Rizosphaera*, query 100%, ident. 82% | KX147946 |
| PO.2.61 | unidentified | *Sarcinomyces* / *Rizosphaera*, query 100%, ident. 82% | KX147947 |
| PO.2.62 | unidentified | *Septorioides*, query 100%, identity 80% | KX147948 |
| PO.2.63 | unidentified | *Sarcinomyces* / *Rizosphaera*, query 100%, ident. 82% | KX147949 |
| PO.2.64 | *Cladosporium* | - | KX147566* |
| PO.2.65 | unidentified | *Sarcinomyces* / *Rizosphaera*, query 100%, ident. 82% | KX147950 |
| PO.2.66 | unidentified | (*Neo*)*catenulostroma*, query 100%, ident. 95% | KX147951 |
| PO.2.67 | unidentified | *Penidiella*, query 28%, identity 84% | KX147952 |
| PO.2.68 | unidentified | *Sarcinomyces* / *Rizosphaera*, query 100%, ident. 82% | KX147953 |
| PO.2.69 | unidentified | *Sarcinomyces* / *Rizosphaera*, query 100%, ident. 82% | KX147954 |
| PO.2.70 | unidentified | (*Neo*)*catenulostroma*, query 100%, ident. 94% | KX147955 |
| PO.2.71 | unidentified | *Sarcinomyces* / *Rizosphaera*, query 100%, ident. 82% | KX147956 |
| PO.2.72 | *Exophiala* | - | KX147957 |
| PO.2.77 | unidentified | *Sarcinomyces* / *Rizosphaera*, query 100%, ident. 82% | KX147958 |
| PO.2.79 | *Exophiala* | - | KX147959 |
| PO.2.81 | unidentified | *Sarcinomyces* / *Rizosphaera*, query 100%, ident. 82% | KX147960 |
| PO.2.82 | unidentified | *Penidiella*, query 28%, identity 84% | KX147961 |
| PO.2.83 | *Lapidomyces* | - | KX147962 |
| PO.2.84 | unidentified | *Penidiella*, query 28%, identity 84% | KX147963 |
| PO.2.85 | unidentified | *Sarcinomyces* / *Rizosphaera*, query 100%, ident. 82% | KX147964 |
| PO.2.86 | *Exophiala* | - | KX147965 |
| PO.2.87 | unidentified | *Sarcinomyces* / *Rizosphaera*, query 100%, ident. 82% | KX147966 |
| PO.2.88 | *Lapidomyces* | - | KX147967 |
| PO.2.78 | *Aureobasidium* | - | KT693498 |
| PO.3.1 | unidentified | *Sarcinomyces* / *Rizosphaera*, query 100%, ident. 82% | KX147968 |
| PO.3.2 | unidentified | *Sarcinomyces* / *Rizosphaera*, query 100%, ident. 82% | KX147969 |
| PO.3.3 | *Lapidomyces* | - | KX147970 |
| PO.3.4 | *Exophiala* | - | KX147971 |
| PO.3.6 | *Lapidomyces* | - | KX147972 |
| PO.3.7 | *Lapidomyces* | - | KX147973 |
| PO.3.8 | unidentified | *Septorioides*, query 100%, identity 81% | KX147974 |
| PO.3.9 | unidentified | *Sarcinomyces* / *Rizosphaera*, query 100%, ident. 82% | KX147975 |
| PO.3.11 | unidentified | *Septorioides*, query 100%, identity 81% | KX147976 |
| PO.3.12 | unidentified | *Sarcinomyces* / *Rizosphaera*, query 100%, ident. 82% | KX147977 |
| PO.3.16 | unidentified | *Septorioides*, query 100%, identity 81% | KX147978 |
| PO.3.17 | unidentified | *Exophiala*, query 100%, identity 96% | KX147979 |
| PO.3.18 | unidentified | *Sarcinomyces* / *Rizosphaera*, query 100%, ident. 82% | KX147980 |
| PO.3.20 | unidentified | *Rhizosphaera*, query 80%, identity 87% | KX147981 |
| PO.3.21 | unidentified | *Sarcinomyces* / *Rizosphaera*, query 100%, ident. 82% | KX147982 |
| PO.3.23 | unidentified | *Sarcinomyces* / *Rizosphaera*, query 100%, ident. 82% | KX147983 |
| PO.3.27 | *Lapidomyces* | - | KX147984 |
| PO.3.28 | unidentified | *Sarcinomyces* / *Rizosphaera*, query 100%, ident. 82% | KX147985 |
| PO.3.31 | unidentified | *Sarcinomyces* / *Rizosphaera*, query 100%, ident. 82% | KX147986 |
| PO.3.32 | unidentified | *Sarcinomyces* / *Rizosphaera*, query 100%, ident. 82% | KX147987 |
| PO.3.33 | unidentified | *Sarcinomyces* / *Rizosphaera*, query 100%, ident. 82% | KX147988 |
| PO.3.34 | *Exophiala* | - | KX147989 |
| PO.3.35 | unidentified | *Sarcinomyces* / *Rizosphaera*, query 100%, ident. 82% | KX147990 |
| PO.3.37 | unidentified | *Sarcinomyces* / *Rizosphaera*, query 100%, ident. 82% | KX147991 |
| PO.3.38 | *Lapidomyces* | - | KX147992 |
| PO.3.39 | unidentified | *Exophiala*, query 100%, identity 96% | KX147993 |
| PO.3.41 | *Lapidomyces* | - | KX147994 |
| PO.3.42 | unidentified | *Septorioides*, query 100%, identity 81% | KX147995 |
| PO.3.43 | *Lapidomyces* | - | KX147996 |
| PO.3.44 | unidentified | *Sarcinomyces* / *Rizosphaera*, query 100%, ident. 82% | KX147997 |
| PO.3.47 | unidentified | *Lapidomyces*, query 64%, ident. 95% | KX147998 |
| PO.3.49 | unidentified | *Sarcinomyces* / *Rizosphaera*, query 100%, ident. 82% | KX147999 |
| PO.3.50 | unidentified | *Sarcinomyces* / *Rizosphaera*, query 100%, ident. 82% | KX148000 |
| PO.3.54 | unidentified | *Sarcinomyces* / *Rizosphaera*, query 100%, ident. 82% | KX148001 |
| PO.3.55 | *Lapidomyces* | - | KX148002 |
| PO.3.57 | unidentified | *Sarcinomyces* / *Rizosphaera*, query 100%, ident. 82% | KX148003 |
| PO.3.58 | unidentified | *Sarcinomyces* / *Rizosphaera*, query 100%, ident. 82% | KX148004 |
| PO.3.60 | *Lapidomyces* | - | KX148005 |
| PO.3.61 | *Lapidomyces* | - | KX148006 |
| PO.3.62 | unidentified | *Sarcinomyces* / *Rizosphaera*, query 100%, ident. 82% | KX148007 |
| PO.3.63 | unidentified | *Sarcinomyces* / *Rizosphaera*, query 100%, ident. 82% | KX148008 |
| PO.3.64 | unidentified | *Phaeococcomyces*, query 96%, identity 95% | KX148009 |
| PO.3.65 | unidentified | *Sarcinomyces* / *Rizosphaera*, query 100%, ident. 82% | KX148010 |
| PO.3.66 | unidentified | *Sarcinomyces* / *Rizosphaera*, query 100%, ident. 82% | KX148011 |
| PO.3.67 | unidentified | *Sarcinomyces* / *Rizosphaera*, query 100%, ident. 82% | KX148012 |
| PO.3.70 | unidentified | *Phaeococcomyces*, query 96%, identity 94% | KX148013 |
| PO.3.71 | unidentified | *Sarcinomyces* / *Rizosphaera*, query 100%, ident. 82% | KX148014 |
| PO.3.72 | *Lapidomyces* | - | KX148015 |
| PO.3.73 | *Lapidomyces* | - | KX148016 |
| PO.3.74 | *Lapidomyces* | - | KX148017 |
| PO.3.75 | unidentified | *Sarcinomyces* / *Rizosphaera*, query 100%, ident. 82% | KX148018 |
| PO.3.76 | *Lapidomyces* | - | KX148019 |
| PO.3.79 | *Lapidomyces* | - | KX148020 |
| PO.3.82 | unidentified | *Sarcinomyces* / *Rizosphaera*, query 100%, ident. 82% | KX148021 |
| PO.3.83 | unidentified | *Phaeococcomyces*, query 96%, identity 95% | KX148022 |
| PO.3.84 | unidentified | *Penidiella*, query 100%, identity 92% | KX148023 |
| PO.3.85 | unidentified | *Phaeococcomyces*, query 96%, identity 95% | KX148024 |
| PO.3.86 | unidentified | *Sarcinomyces* / *Rizosphaera*, query 100%, ident. 82% | KX148025 |
| PO.3.05 | *Aureobasidium* | - | KT693499 |
| PO.3.24 | *Aureobasidium* | - | KT693500 |
| PO.3.68 | *Aureobasidium* | - | KT693501 |
| PO.3.69 | *Aureobasidium* | - | KT693502 |
| PO.3.81 | *Aureobasidium* | - | KT693503 |
| PO.3.88 | *Aureobasidium* | - | KT693504 |

* Sequences were trimmed to remove chimeric parts
